# Volume Change of Masticatory Muscles by Skeletal Class III Orthognathic Surgery with and Without Facial Asymmetry: A Cross-Sectional and Longitudinal Study

**DOI:** 10.3390/jcm15093308

**Published:** 2026-04-26

**Authors:** Hidehito Arima, Chie Tachiki, Takashi Takaki, Keiichi Nishikawa, Tazuko K. Goto, Kenji Sueishi, Yasushi Nishii

**Affiliations:** 1 Department of Orthodontics, Tokyo Dental College, 2-9-18 Kandamisaki-cho, Chiyoda-ku, Tokyo 101-0061, Japan; hidehitoarima@gmail.com (H.A.);; 2 Department of Oral and Maxillofacial Surgery, Tokyo Dental College, 2-9-18 Kandamisaki-cho, Chiyoda-ku, Tokyo 101-0061, Japan; 3 Department of Chemistry, Tokyo Dental College, 2-9-18 Kandamisaki-cho, Chiyoda-ku, Tokyo 101-0061, Japan; 4 Department of Oral and Maxillofacial Radiology, Tokyo Dental College, 2-9-18 Kandamisaki-cho, Chiyoda-ku, Tokyo 101-0061, Japan

**Keywords:** masticatory muscle, muscle volume, computed tomography, three-dimensional reconstruction

## Abstract

**Objectives:** This study aimed to analyze longitudinal changes in masticatory muscle volume at pretreatment and after orthognathic surgery in skeletal Class III patients with and without facial asymmetry. **Methods:** Patients were divided into symmetry and asymmetry groups (*n* = 30, each; male: female ratio 1: 1, for both groups; age at T1: asymmetry group 25.4 ± 8.1, symmetry group 22.9 ± 7.7). Computed tomography images were obtained at pretreatment (T1), one month after surgery (T2), and at 22 months postoperatively (T3). Three-dimensional reconstruction software was used to measure the volumes of the masseter, temporalis, medial pterygoid, and lateral pterygoid muscles. **Results:** At T1, the asymmetry group exhibited significantly smaller masseter and temporalis muscle volumes on the deviated side. These differences tended to decrease at T3. No significant bilateral differences were observed in the medial or lateral pterygoid muscles at each point. Longitudinal analyses showed that temporalis and lateral pterygoid muscle volumes decreased from T1 to T2 and increased from T2 to T3, whereas masseter muscle volume did not have a significantly longitudinal difference and the medial pterygoid muscle volume decreased significantly at T3. **Conclusions:** In Class III patients with facial asymmetry, pretreatment asymmetry of the masseter and temporalis muscle volumes tended to improve postoperatively as bilateral muscle volume increased.

## 1. Introduction

Three-dimensional (3D) analysis makes it easy to gain a 3D understanding of the maxillofacial structure in skeletal Class III patients with facial asymmetry. There have been many reports on skeletal morphology of postoperative changes, which have served as references for treatment improvement via simulations. However, there are fewer reports on masticatory muscles morphology than on skeletal morphology. Traditionally, muscle lengths and cross-sectional areas have been used to ascertain the morphology of masticatory muscles [1,2,3,4]. The ability to make volume comparisons with advances in 3D technology has also made it possible to investigate relationships among masticatory muscles volume, occlusal force, and maxillofacial skeletal structure in skeletal maxillary protrusion and skeletal mandibular prognathism [5,6,7,8,9,10,11]. Therefore, measuring the masticatory muscles volume would be of value in considering their function.

However, there are only a small number of reports comparing the muscle volume of the multiple muscles of mastication in skeletal Class III patients with facial asymmetry. In one report [12], masticatory muscles volume was measured in skeletal Class III patients with facial asymmetry. The masseter on the deviated side was significantly smaller, but no statistically significant differences were observed for the medial or lateral pterygoid muscles. In another report [13], with measurements including the temporalis muscle, it was noted that the medial pterygoid muscle on the non-deviated side was smaller than the deviated side, and no statistically significant differences were observed for the masseter, lateral pterygoid, or the temporalis muscles. Both reports made little use of clinical measurement points of the maxillofacial structure, left unclear the methods used to determine either maxillomandibular relationships or the midsagittal plane, had volume measurements only at pretreatment, and did not investigate changes over time before treatment and after surgical orthodontic treatment. A different report that did track changes in masticatory muscles volume before treatment and after surgical orthodontic treatment only focused on the masseter [14].

The purpose of this study was to measure the maxillofacial structure and masticatory muscles volume in skeletal Class III patients with facial asymmetry, for (1) comparison of pretreatment masticatory muscles volume, (2) comparison of postoperative masticatory muscles volume, and (3) assessment of changes in masticatory muscles volume over time.

## 2. Materials and Methods

This retrospective cohort study was approved by the ethics committee of Tokyo Dental College (approval number: 825 10 October 2017). We performed sample size calculation using G*Power (version 3.1.9.2; Franz Faul, Christian-Albrechts-Universitat, Kiel, Germany). The sample size was calculated for the application of a one-way analysis of variance (ANOVA) for repeated measurements (within-subjects ANOVA for correlated samples). The following parameters were used: effect size F = 0.25; α = 0.05 (5% error), power = 0.80; number of groups = 1; number of measures = 3; correlation between repeated measures = 0.50; and non-sphericity correction = 1. The minimal sample was estimated to include 28 patients per group. Considering the central limit theorem in statistics and the result of the power analysis, the final sample comprised 30 patients per group.

Materials were digital imaging and communications in medicine (DICOM) data for orthognathic surgery between 1 April 2002 and 31 March 2017 at Tokyo Dental College Chiba Hospital. From all patients diagnosed with skeletal mandibular prognathism with or without facial asymmetry, we selected those who underwent orthognathic surgery involving Le Fort I osteotomy and sagittal split ramus osteotomy with plate fixation.

The criteria of facial asymmetry were defined by the Ricketts’ analysis and the definition of the American Association of Oral and Maxillofacial Surgeons [15]: In the frontal cephalogram, less than a 3 mm distance between the midsagittal plane and menton was classified into the symmetry group, and 3 mm or more was classified into the asymmetry group. In the lateral cephalogram, we checked that all patients had finished mandibular growth before capturing computed tomography (CT) data.

**Figure 1:**
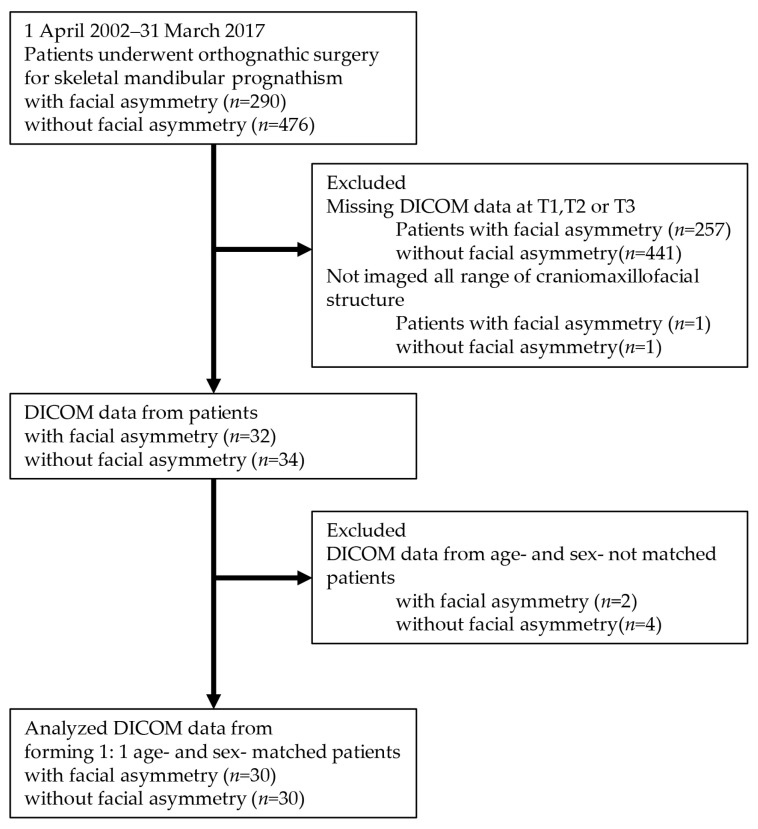
Study flow diagram.

Additionally, we selected DICOM data timing which was captured at pretreatment for diagnosis (T1), at 1 month after surgery for evaluating orthognathic surgery (T2) and at 6 months or more after surgery for postoperative genioplasty or for plate removal (T3). Table 1 shows patients’ age at T1 and the term from operation date to T2 or T3. The age and gender affect muscle volume [16,17,18]. Thirty people were selected for the asymmetry group (15 male and 15 female; age at T1: 15.0 to 45.8 years; mean: 25.4 ± 8.1 years). After that, we selected 30 age- and sex-matched subjects for the symmetry group (15 male and 15 female; age at T1: 15.6 to 47.9 years; mean: 22.9 ± 7.7 years). The exclusion criteria were as follows: if the causes of facial asymmetry were symptomatic diseases, trauma, soft tissue, temporomandibular ankyloses, neoplasm and allied diseases, condylar hyperplasia, and functional deviation; if DICOM data were missing at T1, T2 or T3, and if the range of craniomaxillofacial structure not all imaged; if there were genioplasty or other additional procedures with orthognathic surgery (Figure 1).

### 2.1. Measuring Devices

The CT scans were obtained with the Siemens SOMATOM Volume Zoom or SOMATOM Definition AS (Siemens Healthcare: Erlangen, Germany). All patients were asked to bite gently with lips closed, while their heads were fixed with specially made ear rods to standardize their position during the CT scan. Table 2 shows differences regarding imaging parameters. The two scanners were set to be as similar as possible, and adjustments were made to reduce the partial volume effect, so that the accuracy difference between the scanners would be minimized. All DICOM data were imported in SimPlant OMS software (Dental Version 14.0: Materialise Software, Leuven, Belgium) for analyzing measurements and 3D volume rendering. 3D models of muscles of mastication were reconstructed by tracing with a digitizer (Cintiq 27QHD touch, Wacom, Saitama, Japan).

### 2.2. Measurement Methods

#### 2.2.1. Maxillofacial Structure

Table 3 shows the details of the measurements. We set the Frankfort horizontal (FH) plane and the midsagittal plane according to a report from An et al. [19]. We used two midsagittal planes for evaluating facial asymmetry. One plane was passed through nasion (N) and basion (Ba) and was perpendicular to the FH plane for reproducibility. The other plane passed through N, Ba, and the anterior nasal spine (ANS) to consider the maxilla lateral deviation. We again measured the amount of menton (Me) lateral deviation at the CT images. Since some cases also had undergone genioplasty at T2 or T3, we also measured the amount of lateral deviation at the mental spine (MeS), which would be unaffected. 3D measurements were created according to the Downs–Northwestern method.

In the asymmetry group, individuals with the first molar on the deviated side exhibiting crossbite (CB) were placed in a CB group. Those not exhibiting CB were placed in a normal bite (NB) group, based on the report from Suzuki et al. [20], and they were assigned to a bilateral Class III group and unilateral Class III group (Table 1).

#### 2.2.2. Masticatory Muscles Volume

The investigator measured the 3D masticatory muscles volume, which was constructed by tracing. The 3D masticatory muscles consist of the 3D masseter, temporalis, medial pterygoid, and lateral pterygoid muscles. For determining the boundaries of each of the masticatory muscles during tracing, the image was magnified enough to make the boundaries discernible (4× to 9×). Tracing was done in a horizontal cross-section, with confirmation and correction by reference to sagittal and coronal cross-sections, followed by correction again in the horizontal cross-section. The resulting 3D structure was again checked and corrected as necessary. For normal anatomy of the masticatory muscles, we referred to the ranges of attachment and normal anatomy from reports [21,22,23,24].

**Figure 2:**
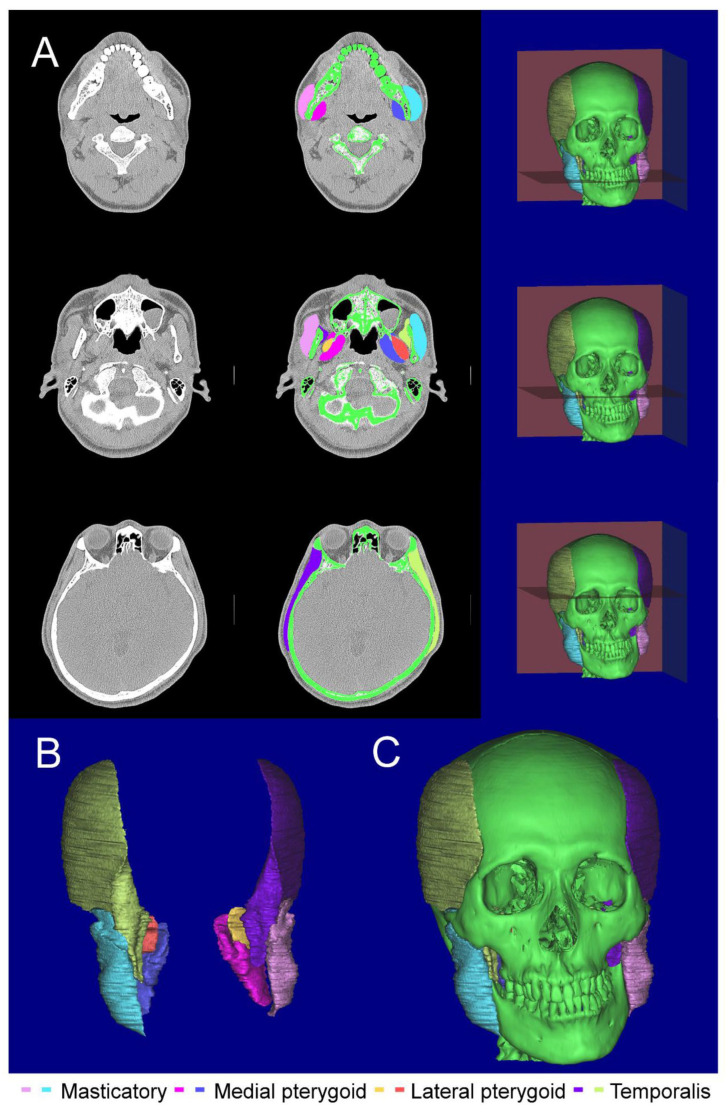
Tracing and 3D model of masticatory muscles and maxillofacial structure. (**A**) Tracing in a typical horizontal cross-section; (**B**) 3D models of masticatory muscles; (**C**) 3D model of masticatory muscles and maxillofacial structure.

The masseter muscle includes superficial, middle, and deep layers. The temporalis muscle excluded portions arising from the fascia. The medial pterygoid muscle included superficial and deep heads, and the lateral pterygoid muscle included upper and lower heads. The investigator prepared by dissecting the masticatory muscles from donated cadavers and thoroughly practicing tracing; an anatomy specialist checked the resulting 3D structure, thus creating elaborate 3D models of the masticatory muscles (Figure 2). Regarding reproducibility, the Pearson correlation coefficient according to the test–retest method (the masseter muscle was traced to build a 3D model five consecutive times, with the same method being carried with a duration of one month) was 0.98, and the intraclass correlation coefficient (ICC) was ICC (1,1) = 0.913, which was considered meticulous and precise.

### 2.3. Statistical Analysis

Statistical analysis was performed using SPSS software (version 24; IBM, Armonk, NY, USA). We tested the normal distribution using the Shapiro–Wilk test and all measurements showed a normal distribution.

In the symmetry group, mandibular plane and masticatory muscles volume were analyzed by paired *t*-test. No statistically significant differences were observed between the right and left sides. A *t*-test was performed for the measurements of maxillofacial structure and masticatory muscles volume, to determine differences asymmetry group. A paired *t*-test was performed for the differences between the deviated side and the non-deviated side in each of the masticatory muscles at the respective times T1, T2, and T3. When there were significant differences between the deviated side and the non-deviated side at T1 in the asymmetry group, we tested the effect size (Cohen d) at T1 and T3 to examine how the differences changed at T3. Bonferroni correction was used to compensate for multiple comparisons. To examine how muscle volume changed over time, a repeated-measures one-way ANOVA was performed for masticatory muscles volume at T1, T2, and T3, followed by a paired *t*-test with Bonferroni correction.

## 3. Results

### 3.1. Maxillofacial Structure

The amounts of both anterior and lateral skeletal deviation of the mandible displayed a significant difference at T1 between the symmetry and the asymmetry groups. The symmetry group had anterior deviation of the mandible and little lateral deviation, while the asymmetry group had lateral deviation of the mandible and little anterior deviation. No significant differences were observed in the maxillary measurements. In the measurements of teeth, the only significant difference was in the amount of lateral deviation, with the asymmetry group having significantly greater midline discrepancy.

For anteroposterior molar occlusion, the symmetry group all had Class III on both sides, while half of the asymmetry group had Class III on both sides. For lateral molar occlusion, the majority of the symmetry group had bilateral normal bite occlusion, while the majority of the asymmetry group had crossbite on the deviated side (Table 1). At T2, the asymmetry group had significantly more lateral deviation in Me, but no significant difference was observed in MeS. The genioplasty eliminated lateral Me deviation at T3. At T2 and T3, the symmetry group had significantly more anterior mandibular deviation. Due to the difference in the number of Class III molar relationships between the symmetry and asymmetry group, there was a significant difference in treatment results. There was no skeletal deviation at T3. Due to the shifting of the teeth, no statistically significant differences were observed in the mandibular plane; OB distance was significantly smaller in the asymmetry group (Table 4).

### 3.2. Comparison of Masticatory Muscles Volume at Pretreatment

**Figure 3:**
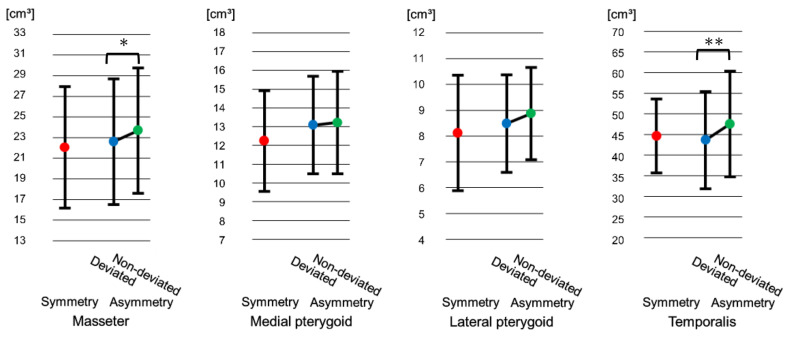
Comparison of masticatory muscles volume at pretreatment (T1). Error bar indicates standard deviation. * *p* < 0.05, ** *p* < 0.01. (Bonferroni adjustment applied). On the deviated side, the masseter and temporalis muscles volume were significantly smaller in the asymmetry group.

No statistically significant differences were observed at T1 between the symmetry and the asymmetry groups in masticatory muscles volume. However, there was a significant difference in the masseter and temporalis muscles volume between the deviated and the non-deviated sides in the asymmetry group, where muscle volume was significantly smaller in the deviated side for both muscles (masseter: *p* < 0.05, temporalis: *p* < 0.01). No significant difference was observed in the medial and lateral pterygoid muscles in the asymmetry group between the deviated and the non-deviated sides (medial pterygoid: *p* = 0.46, lateral pterygoid: *p* = 0.12) (Figure 3).

### 3.3. Comparison of Postoperative Masticatory Muscles Volume

**Figure 4:**
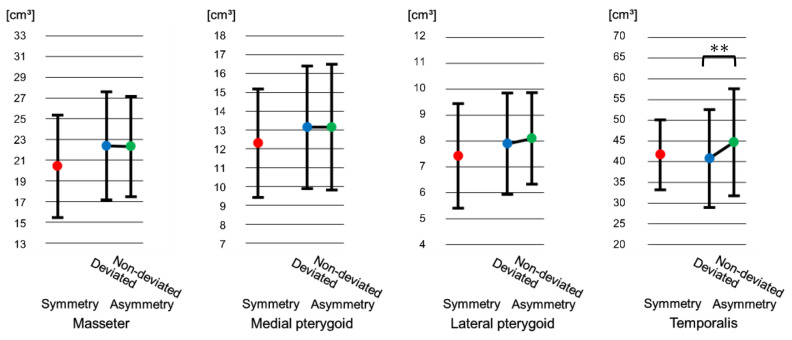
Comparison of postoperative masticatory muscles volume at 1 month (T2). Error bar indicates standard deviation. ** *p* < 0.01. (Bonferroni adjustment applied). On the deviated side, the temporalis muscle volume was significantly smaller in the asymmetry group.

At T2, no statistically significant differences were observed between the symmetry and the asymmetry groups in masticatory muscles volume. The temporalis muscle had significantly less volume in the deviated side of the asymmetry group (*p* < 0.01). No significant difference was observed in the masseter, medial pterygoid, and lateral pterygoid muscles of the asymmetry group between the deviated and the non-deviated sides (masseter: *p* = 0.88, medial pterygoid: *p* = 0.99, lateral pterygoid: *p* = 0.41) (Figure 4).

**Figure 5:**
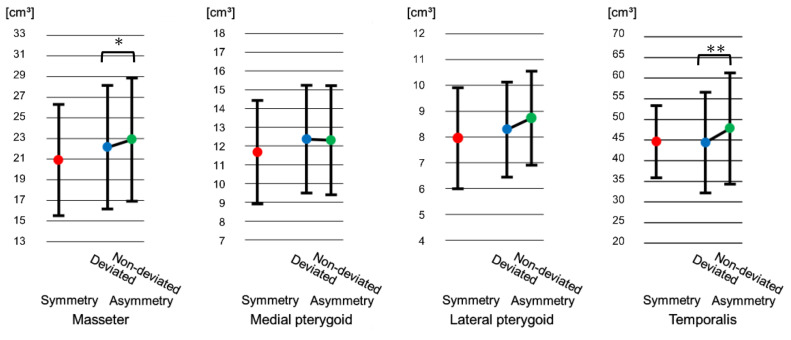
Comparison of postoperative masticatory muscles volume at 21 months (T3). Error bar indicates standard deviation. * *p* < 0.05, ** *p* < 0.01. (Bonferroni adjustment applied). On the deviated side, the masseter and temporalis muscles volume were significantly smaller in the asymmetry group.

At T3, no statistically significant differences were observed between the symmetry and the asymmetry groups in masticatory muscles volume. However, the asymmetry group had a significant difference in the masseter and temporalis muscles between the deviated and the non-deviated sides, with the deviated side being significantly smaller than the non-deviated side for both muscles (masseter: *p* < 0.05, medial pterygoid: *p* = 0.80, lateral pterygoid: *p* = 0.07, temporalis: *p* < 0.01) (Figure 5).

At T1 and T3, masseter and temporalis muscles in the asymmetry group with the deviated side were both smaller than in the non-deviated side. The comparison of differences between the deviated and the non-deviated sides in the masseter and temporalis muscles volume at T1 and T3 in the asymmetry group showed that the effect size at T1 (masseter: d = 0.46; temporalis: d = 0.96) decreased at T3 (masseter: d = 0.38; temporalis: d = 0.90). Therefore, those differences at T1 had decreased at T3.

### 3.4. Assessment of Changes in Masticatory Muscles Volume over Time

#### 3.4.1. Symmetry Group

**Figure 6:**
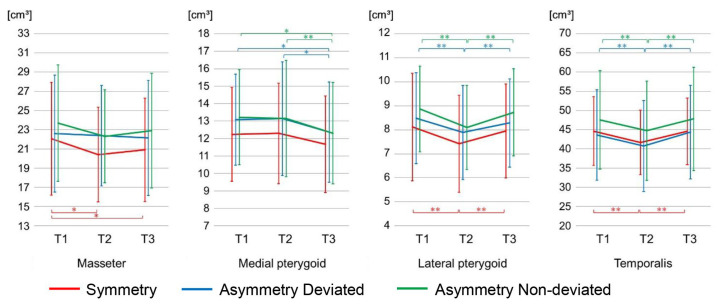
Changes over time in masticatory muscles volume. Error bar indicates standard deviation. * *p* < 0.05, ** *p* < 0.01. (Bonferroni adjustment applied). Asymmetry of pretreatment masseter and temporalis muscles volume approaches symmetry as the bilateral postoperative volume increases.

Although the lateral pterygoid and temporalis muscles volume decreased significantly from T1 to T2 (lateral pterygoid: *p* < 0.01, temporalis: *p* < 0.01), and then increased significantly from T2 to T3 (lateral pterygoid: *p* < 0.01, temporalis: *p* < 0.01), no statistically significant differences were observed between T1 and T3 (lateral pterygoid: *p* = 0.82, temporalis: *p* = 1.00). The masseter muscle volume decreased significantly from T1 to T2 (*p* < 0.05), and tended to increase from T2 to T3 (*p* = 0.35), but remained reduced compared with T1 (*p* < 0.05). With the medial pterygoid muscle, no statistically significant differences were observed among volume at T1, T2, and T3 (T1T2: *p* = 1.00, T2T3: *p* = 0.20 T1T3: *p* = 0.20) (Figure 6).

#### 3.4.2. Asymmetry Group

Although the lateral pterygoid and temporalis muscles volume decreased significantly from T1 to T2 (*p* < 0.01), and increased significantly from T2 to T3 (*p* < 0.01), there was found to be no significant difference between T1 and T3 (lateral pterygoid; deviated: *p* = 0.42; non-deviated: *p* = 0.96, temporalis; deviated: *p* = 0.88; non-deviated: *p* = 1.00). The masseter muscle volume did not show a significant volume difference among T1, T2, and T3 (deviated; T1T2: *p* = 1.00; T2T3: *p* = 1.00; T1T3: *p* = 0.87, non-deviated T1T2: *p* = 0.08; T2T3: *p* = 0.90; T1T3: *p* = 0.34); the medial pterygoid muscle volume had no significant difference at T1 and T2 (*p* = 1.00), then decreased significantly from T2 to T3 (*p* < 0.05), it remained significantly reduced as compared with T1 and T3 (deviated: *p* < 0.05 non-deviated: *p* < 0.01) (Figure 6).

## 4. Discussion

### 4.1. Comparison of Masticatory Muscles Volume at Pretreatment

Our results showed that in skeletal Class III patients with facial asymmetry, the masseter and temporalis muscles volume on the deviated side are significantly smaller than those on the non-deviated side (Figure 3). Kiliaridis et al. [25] reported that in cases of crossbite, the masseter has a smaller cross-sectional area on the crossbite side than on the side not exhibiting crossbite. Suzuki et al. [20] reported that in instances which the deviated side exhibits crossbite in facial asymmetry patients, the deviated side does not demonstrate a normal path of jaw movement, but has a shortened path. In this study, the CB exhibited a rate of 57%, and that might influence the masseter and temporalis muscles on the deviated side had a significantly smaller volume than on the non-deviated side.

Goto et al. [12] reported that the masseter muscle volume on the deviated side is smaller than on the non-deviated side in skeletal Class III patients with facial asymmetry. We considered that the similar result to ours was due to using similar materials and conditions. However, Kwon et al. [13] reported that the medial pterygoid muscle on the non-deviated side is smaller than on the deviated side, which is different from our results. We considered that the difference might be due to the characteristics of the patients. Our facial asymmetry patients had a lesser degree of mandibular prognathism, 50% exhibited unilateral Class I with a mean ANB of −0.02°, therefore, Kwon et al. might have analyzed the data of patients with facial asymmetry having a greater degree of mandibular prognathism.

### 4.2. Comparison of Postoperative Masticatory Muscles Volume

In the asymmetry group, the volume differences of the deviated and the non-deviated side on the masseter and temporalis muscles at T1 tended to decrease at T3 (Figure 3, Figure 4 and Figure 5). We considered that surgical orthodontic treatment affected not only maxillofacial structure and dental arch symmetry but also masticatory muscles symmetry. Seo et al. [14] measured masseter muscle volume at pretreatment and 1 year after surgery in skeletal Class III patients with facial asymmetry which were treated both a Le Fort I and intraoral vertical ramus osteotomy (IVRO). They reported that the masseter muscle volume on the deviated side significantly increased from pretreatment to 1 year after surgery. There were some differences that must be taken into consideration between that report and ours; however, masticatory muscles volume in skeletal Class III patients with facial asymmetry tends to become symmetrical after surgery by increasing volume.

Eo et al. [26] measured masticatory muscle volume at pretreatment and after surgery in skeletal Class III patients with facial asymmetry who were treated both a Le Fort I and SSRO. They reported that the difference in masseter muscle volume on both sides was significantly reduced after surgery due to the tendency of decreased volume on the non-deviated side. While the reduction in asymmetry after surgery is similar to our result, whether that is due to an increase or decrease in masticatory muscle volume, may be influenced by differences in the amount of bone movement during surgery and the postoperative treatment.

### 4.3. Assessment of Changes in Masticatory Muscles Volume over Time

#### 4.3.1. Temporalis Muscle and Lateral Pterygoid Muscle

The reason for the decrease from T1 to T2 may be that the function of the masticatory muscles was reduced by dental decompensation in presurgical orthodontic treatment. The reducing masticatory muscles’ function might influence the volume and remained at T2. The reason for the increase from T2 to T3 may be that the orthognathic surgery and postoperative orthodontic treatment resulted in normal occlusion, influencing a recovery in masticatory function, therefore increasing muscle volume again.

Choi et al. [27] reported that in patients with skeletal mandibular prognathism who underwent a Le Fort I osteotomy and IVRO, there was a decrease in occlusal contact area and occlusal force from pretreatment to immediately before surgery. The greatest decrease was at 1 month after surgery, and an increase from 1 month after surgery to 2 years after surgery. In their discussion, they stated that dental decompensation was responsible for the decrease in occlusal force and occlusal contact area from pretreatment to 1 month after surgery, and that the orthognathic surgery and postoperative orthodontic treatment were responsible for the increase from 1 month after surgery to 2 years after surgery, which supports our results.

#### 4.3.2. Masseter Muscle

In the asymmetry group, the lack of decrease from T1 to T2 may be influenced by more subjects, such as a lifting of the site of the masseter attachment off the angle of the mandible, and possibly an inflammatory reaction resulting in postoperative swelling at T2 causing an increase in masseter muscle volume at T2. These may have masked a decrease in volume from T1 to T2 [28,29]. The lack of increase from T2 to T3 may be similarly influenced by the effects of swelling at T2. The reason that no statistically significant differences were observed between T1 and T3, unlike the masseter muscle volume in the symmetry group, may be that the asymmetry group at pretreatment had more crossbite, and thus they had more recovery of masticatory function and increasing the volume as compared to the symmetry group.

#### 4.3.3. Medial Pterygoid Muscle

Regarding the medial pterygoid muscle volume in the symmetry group, an increase at T2 may be due to remaining swelling at the time of the orthognathic surgery, which is also considered to mask the decrease from T1 to T2. The reason is that swelling, which has less of an effect on the masseter muscle volume in the symmetry group, might cause detachment and injury to this muscle. The masking of the increase from T2 to T3 may be similarly due to the effects of swelling at T2. No significant difference between T1 and T3 may also be influenced by recovery of masticatory function.

Meanwhile, regarding the medial pterygoid muscles of the asymmetry group, an increase in volume at T2 might be due to swelling masking a decrease from T1 to T2. In the asymmetry group, the removal of bone at the attachment of the medial pterygoid muscle for preventing relapse might influence the decrease in the asymmetry group from T2 to T3 and the decrease from T1 to T3, in contrast to the symmetry group. The asymmetry group underwent removal of the bone at the attachment of the medial pterygoid muscle to prevent relapse when the distal bone fragment was being positioned. Therefore, the medial pterygoid muscles in the asymmetry group might have been injured during the orthognathic surgery, thus could not fully recover [30], which might result in a volume decrease.

### 4.4. Limitations

In this study, the postoperative range of T3 followed a normal distribution, but it is larger than the range of T1 and T2, suggesting that postoperative orthodontics and long-term remodeling may be influencing the results. Furthermore, this study contains a mix of posterior relationships (Class I, Class III) and crossbite patterns (NB, CB), which may have influenced the results.

We could not determine from the CT images whether the bone and muscle were firmly attached or merely adjacent. Establishing a technique for discerning muscle attachment would therefore make it possible to show precisely whether the extent of muscle detachment and injury during surgery affects muscle volume.

Additionally, we were not able to examine function. In the future, we will examine the relationship between function and volume of each masticatory muscle in detail, which will enable us to understand the postoperative muscle condition more clearly.

### 4.5. Clinical Implications

Similar to skeletal muscles of the limbs, the recovery of intraoral function caused an increase at the cross-sectional area of masticatory muscles after orthodontic surgery [31,32]. Thus, we considered that rehabilitation on the deviated side might increase the masseter and temporalis muscle volume on the deviated side. Furthermore, the difference between the deviated side and the non-deviated side might decrease after surgery.

## 5. Conclusions

In skeletal Class III patients, the presence of facial asymmetry did not significantly affect the volume of each masticatory muscle.

However, in skeletal Class III patients with facial asymmetry, the volumes of masseter and temporalis muscles were significantly smaller on the deviated side than the non-deviated side, and no significant differences were observed in the medial and lateral pterygoid muscles on both sides.

Postoperatively, the asymmetry in masseter and temporalis muscle volumes persisted, although the differences tended to be less pronounced compared with those at pretreatment.

In skeletal Class III patients with facial asymmetry, asymmetry of the masseter and temporalis muscles volume approaches symmetry as the bilateral postoperative volume increases.

These findings may contribute to a better understanding of masticatory muscle adaptation and postoperative changes in patients with facial asymmetry.

## Figures and Tables

**Table 1:** Patient details.

		**Symmetry (*n* = 30)**	Asymmetry (*n* = 30)		*p* value
**Sex**		**15 males, 15 females**	15 males, 15 females		
Age at T1 (y)	Mean ± SD	22.9 ± 7.7	25.4 ± 8.1		NS
	Range	15.6–47.9	15.0–45.8		NS
T2—operation day (m)	Mean ± SD	1.2 ± 0.3	1.2 ± 0.5		NS
	Range	0.9–2.4	0.2–2.8		NS
T3—operation day (m)	Mean ± SD	21.7 ± 9.1	21.6 ± 7.8		NS
	Range	9.9–42.0	9.4–37.2		NS
			Deviated	Non-deviated	
Molar relationships Anteroposterior	Class III	100% (60/60)	50% (15/30)	100% (30/30)	
	Class I	0% (0/60)	50% (15/30)	0% (0/30)	
Molar relationships Lateral	NB	63% (38/60)	43% (13/30)	83% (25/30)	
	CB	37% (22/60)	57% (17/30)	17% (5/30)	

NS: Not significantly difference.

**Table 2:** Imaging parameters.

	Volume Zoom	Definition AS
Visual field for imaging	230 mm diameter	230 mm diameter
Tube voltage	120 kV	120 kV
X-ray dose	88 mAs	88 mAs
Scan speed	1 s/rotation	1 s/rotation
Slice thickness	1.0 mm	1.0 mm
Slice interval	1.0 mm	1.0 mm
Reconstruction algorithm	H60s	H60s
Pixel size	0.44 mm	0.44 mm
Beam width	1 mm × 4	0.6 mm × 32
Beam pitch	3 mm/rotation	17.6 mm/rotation

**Table 3:** Landmarks, lines, planes, and measurements used in the study.

Landmark/line/plane	Abbreviation	Definition
Average gonion	Go	Midpoint of gonion of both sides
Average orbitale	Or	Midpoint of orbitale of both sides
Average porion	Po	Midpoint of porion of both sides
Mental Spine	MeS	Midway between two pairs of peaks of the inferior mental spine, distal end if fused
Upper incisors point	IsU1	Midpoint of maxillary central incisors of both sides
Lower incisors point	IsL1	Midpoint of mandibular central incisors of both sides
Incisors point	U1L1	Midpoint between IsU1 and IsL1
PoLOrL(PoROrR) line	PoLOrL(PoROrR)	Line between porion left (right) and orbitale left (right)
PoOr line	PoOr	Line between Po and Or
GoLMe(GoRMe) line	GoLMe(GoRMe)	Line between GoL(GoR) and Me
GoMe line	GoMe	Line between Go and Me
Frankfort holizontal plane	FH	Plane defined by orbitales of both sides and Po
Dental occlusal plane	DOP	Plane defined by the overbiting points of both sides first molars and U1L1
FHNBa plane	FHNBa	Plane passing through nasion, basion and perpendicular to FH
ANSNBa plane	ANSNBa	Plane defined by ANS, N and Ba
Measurement		
ANS-FHNBa (mm)		Distance between ANS and FHNBa
Me-ANSNBa (mm)		Distance between Me and ANSNBa
Me-FHNBa (mm)		Distance between Me and FHNBa
MeS-FHNBa (mm)		Distance between MeS and FHNBa
SNA (°)		Angle from sella via nasion to A-point
SNB (°)		Angle from sella via nasion to B-point
ANB (°)		Difference of measurement SNA and measurement SNB
Average mandibular plane (°)		Angle between GoMe and PoOr
Deviated side mandibular plane (°)		Angle between PoLOrL and GoLMe (if Me deviated left side)
Non-deviated side mandibular plane (°)		Angle between PoROrR and GoRMe (if Me deviated left side)
Midline discrepancy (mm)		Distance of IsU1 and IsL1 from FHNBa (absolute value)
OJ (mm)		Distance of IsU1 and IsL1 with vertical projection onto DOP (a negative value indicates anterior crossbite)
OB (mm)		Distance of IsU1 and IsL1 from DOP (a negative value indicates open bite)

**Table 4:** Assessment of maxillofacial structure.

	T1						T2						T3					
	Symmetry		Asymmetry				Symmetry		Asymmetry				Symmetry		Asymmetry			
	Mean	SD	Mean	SD	*p* value		Mean	SD	Mean	SD	*p* value		Mean	SD	Mean	SD	*p* value	
ANS-FHNBa (mm)	0.94	0.83	1.37	0.91	0.06	NS	1.64	1.31	1.48	1.34	0.63	NS	1.48	1.26	1.45	1.34	0.92	NS
Me-ANSNBa (mm)	2.11	1.19	9.24	4.17	0.00	**	2.94	2.34	3.96	3.31	0.17	NS	2.47	1.68	3.73	3.06	0.05	NS
Me-FHNBa (mm)	2.15	1.14	10.18	4.81	0.00	**	2.39	2.44	4.26	2.89	0.00	**	2.87	2.65	3.97	2.88	0.13	NS
MeS-FHNBa (mm)	2.26	1.21	8.12	3.82	0.00	**	2.31	2.38	3.18	2.22	0.15	NS	2.48	2.52	3.53	2.65	0.12	NS
SNA (°)	81.60	3.79	80.38	3.86	0.22	NS	81.95	3.46	80.67	3.89	0.18	NS	81.29	3.44	80.20	4.39	0.29	NS
SNB (°)	84.85	3.91	80.39	4.33	0.00	**	80.04	3.93	78.01	3.34	0.03	*	80.76	3.91	78.51	3.65	0.02	*
ANB (°)	−3.25	2.88	−0.02	3.42	0.00	**	1.90	2.46	2.66	2.57	0.25	NS	0.53	2.36	1.69	2.56	0.07	NS
Average mandibular plane (°)	28.50	4.92	30.99	6.47	0.30	NS ^1^	30.69	5.46	31.49	5.74	1.00	NS ^1^	31.03	5.72	31.89	6.21	1.00	NS ^1^
Deviated side mandibular plane (°)			31.95	4.86	1.00	NS ^1^			33.58	4.54	1.00	NS ^1^			33.74	4.44	1.00	NS ^1^
Non-deviated side mandibular plane (°)			33.04	6.36	0.39	NS ^1^			33.44	4.88	1.00	NS ^1^			33.98	5.24	1.00	NS ^1^
Midline discrepancy (mm)	1.87	1.34	4.38	1.85	0.00	**	0.60	0.53	0.51	0.46	0.51	NS	0.61	0.44	0.59	0.56	0.90	NS
OJ (mm)	−1.96	2.83	−0.35	3.73	0.06	NS	3.23	0.97	3.08	0.66	0.48	NS	3.18	0.87	2.89	0.65	0.15	NS
OB (mm)	0.56	2.33	0.58	1.98	0.97	NS	1.63	1.05	1.16	0.83	0.06	NS	1.73	0.96	1.12	0.94	0.02	*

NS: Not significantly different, * *p* < 0.05, ** *p* < 0.01: Statically significant difference. ^1^: * *p* < 0.05, ** *p* < 0.01. Bonferroni adjustment.

## Data Availability

Due to privacy restrictions, all data are stored at the corresponding author institution. Qualified researchers are able to gain access via an application to the corresponding author.
